# Lobular Breast Cancer Conspicuity on Digital Breast Tomosynthesis Compared to Synthesized 2D Mammography: A Multireader Study

**DOI:** 10.3390/jimaging7090185

**Published:** 2021-09-12

**Authors:** Giovanna Romanucci, Lisa Zantedeschi, Anna Ventriglia, Sara Mercogliano, Maria Vittoria Bisighin, Loredana Cugola, Paola Bricolo, Rossella Rella, Marta Mandarà, Chiara Benassuti, Andrea Caneva, Francesca Fornasa

**Affiliations:** 1 UOSD Breast Unit ULSS9, Ospedale di Marzana, Piazzale Lambranzi, 1, 37142 Verona, Italy; anna.ventriglia@aulss9.veneto.it (A.V.); mariavittoria.bisighin@aulss9.veneto.it (M.V.B.); loredana.cugola@aulss9.veneto.it (L.C.); paola.bricolo@aulss9.veneto.it (P.B.); 2 Department of Radiology, G. Fracastoro Hospital, San Bonifacio, 37047 Verona, Italy; lisa.zantedeschi@aulss9.veneto.it (L.Z.); francesca.fornasa@aulss9.veneto.it (F.F.); 3 A Division of Radiology, Ospedale Medico-Chirurgico Accreditato Villa dei Fiori, Acerra, 80011 Naples, Italy; sara.mercogliano90@gmail.com; 4 Department of Diagnostic Imaging, G.B. Grassi Hospital, ASL ROMA 3, Via Gian Carlo Passeroni, 28, 00122 Rome, Italy; rossella.rella@gmail.com; 5 Department of Medical Oncology, ULSS9 Scaligera, G. Fracastoro Hospital, San Bonifacio, 37047 Verona, Italy; marta.mandara@aulss9.veneto.it; 6 UOSD Breast Surgery, ULSS9 Scaligera, G. Fracastoro Hospital, San Bonifacio, 37047 Verona, Italy; chiara.benassuti@aulss9.veneto.it; 7 Division of Pathology, ULSS9 Scaligera, G. Fracastoro Hospital, San Bonifacio, 37047 Verona, Italy; andrea.caneva@aulss9.veneto.it

**Keywords:** digital breast tomosynthesis, invasive lobular carcinoma, breast cancer, conspicuity

## Abstract

Objectives: To compare the conspicuity of lobular breast cancers at digital breast tomosynthesis (DBT) versus synthesized 2D mammography (synt2D). Materials and methods: Seventy-six women (mean age 61.2 years, range 50–74 years) submitted to biopsy in our institution, from 2019 to 2021, with proven invasive lobular breast cancer (ILC) were enrolled in this retrospective study. The participants underwent DBT and synt2D. Five breast radiologists, with different years of experience in breast imaging, independently assigned a conspicuity score (ordinal 6-point scale) to DBT and synt2D. Lesion conspicuity was compared, for each reader, between the synt2D overall conspicuity interpretation and DBT overall conspicuity interpretation using a Wilcoxon matched pairs test. Results: A total of 50/78 (64%) cancers were detected on both synt2D and DBT by all the readers, while 28/78 (26%) cancers where not recognized by at least one reader on synt2D. For each reader, in comparison with synt2D, DBT increased significantly the conspicuity of ILC (*p* < 0.0001). The raw proportion of high versus low conspicuity by modality confirmed that cancers were more likely to have high conspicuity at DBT than synt2D. Conclusions: ILCs were more likely to have high conspicuity at DBT than at synt2D, increasing the chances of the detection of ILC breast cancer.

## 1. Introduction

In recent years, digital breast tomosynthesis (DBT) in combination with synthetized mammography (synt2D) has proved to be an effective imaging technique for the detection of early-stage breast cancer. This seems to be particularly evident for invasive breast cancers, rather than in situ malignancy. Digital breast tomosynthesis is a pseudo-3D technique that allows overcoming tissue superimposition with a consequentially better view of margin analysis compared to conventional mammography. As a result, enhancing the lesion margins improves the evaluation of masses and increases the detection of architectural distortions and asymmetries. This higher sensitivity of DBT for these various types of mammographic findings, especially architectural distortion, improves the visibility of invasive lobular cancers [1]. Invasive lobular carcinoma (ILC) represents the second most common histological type of breast cancer affecting 5%–15% of women with invasive breast cancer. Although in the last two decades there has been a marked increase in the incidence of ILC, mainly among the post-menopausal population, it is still diagnosed with an average age at diagnosis three years older than invasive ductal carcinoma [2]. ILC remains difficult to detect, both upon physical examination, due to the absence of clinical symptoms (as a lump), and on routine mammograms, due to distinctive histopathological hallmarks. Detection of ILC using standard imaging is particularly challenging due to the infiltrative tumor growth pattern that does not destroy the underlying anatomic structures or incite a desmoplastic stromal reaction [2]. There have already been several studies about the usefulness of DBT in breast cancer conspicuity [3,4,5] but not many on the role of DBT in evaluation of the conspicuity of ILC, in particular. The purpose of this study was to assess the conspicuity of ILC on DBT compared to synt2D. Our intention was to show how DBT allows better identification of ILC, increasing the chances of successful treatment.

## 2. Materials and Methods

This retrospective single-center, multi-reader study was conducted with the approval of the institutional review board. All screening patients with ILC submitted to histologic biopsy in our institution, from September 2019 to April 2021, were considered for inclusion in the study (screening mammography).

Inclusion criteria were percutaneous biopsy-proven primary ILC and women who underwent DBT plus synt2D. Women with previous breast surgery and/or history of previous breast cancer were not enrolled. Thus, our study population consisted of 76 patients, with an average age of 61.2 years (range 50–74 years).

Participants underwent a DBT plus synt2D using a Selenia Dimensions Unit (Hologic, Inc., Bedford, MA, USA). Each of the synt2D and 3D acquisitions included cranio-caudal (CC) and mediolateral oblique (MLO) views. Biopsies were performed following standard clinical practice. Digital breast tomosynthesis-guided biopsies were performed on a Selenia Dimensions unit (Hologic, Bedford, MA, USA) using an 9-gauge vacuum-assisted biopsy device. After the DBT procedure, a localizing marker was deployed at each biopsy site, and a two-view mammogram was obtained to document marker deployment and final position. Ultrasound-guided biopsies were obtained using a 14-gauge core needle.

This study involved five breast dedicated radiologists (reader 1, R1; reader 2, R2; reader 3, R3; reader 4, R4; reader 5, R5). They had between 5 and 25 years of experience in diagnostic and/or screening mammography. DBT experience was between 1 and 8 years (with at least 5000 DBT—readings per year). A dedicated work-station with high-spatial-resolution mammography monitors was used to read both synt2D and DBT examinations. The radiologists were completely blinded to any clinical or previous radiological data; however, they knew that all women had proven ILC.

Two reading sessions were performed (separated by at least three weeks to avoid memory bias): in the first reading session, the readers evaluated synt2D exams, in the second, they evaluated DBT exams. In each reading session, they independently assigned a conspicuity score to both CC and MLO views of DBT and 2D-synt. The conspicuity was rated on a 6-point confidence scale (0 = absent, 1 = minimal, 2 = sufficient, 3 = good, 4 = very good, 5 = excellent).

For the purpose of analysis, the conspicuity assignments were subsequently dichotomized as follows: “low” including 0, 1, and 2 values and “high” including 3, 4, and 5 values.

For each lesion another dedicated breast radiology retrospectively analyzed the following qualitative and quantitative data on DBT examinations: breast density (fatty breast A or B density; dense breast C or D density); lesion location (left or right breast); mammographic features of the lesions (opacity, opacity plus calcifications, architectural distortion, asymmetry and calcifications), and maximum diameter of the lesion.

Frequencies and percentages were used to summarize the mammographic and histopathologic features of ILC detected on both synt2D and DBT. In addition, for the subgroup of the cancers identified by all the readers with both the modalities and for the subgroup of anomalies just recalled by all the readers with only 3D-DBT, descriptive statistics (numbers and percentages) were used to describe lesion type and breast density. Lesion conspicuity was compared, for each reader, between synt2D overall conspicuity interpretation and DBT overall conspicuity interpretation using a Wilcoxon matched pairs test. A *p*-value of less than 0.05 was considered to be statistically significant. In order to analyze the percentage difference observed between the two modalities, the average scores from the five readers’ measurements were dichotomized into low (score of 0–2) conspicuity and high (score of 3–5) conspicuity.

For each lesion, tumor size was evaluated at definitive histology and percentages were used to summarize tumor size (pT).

## 3. Results

Our collective of 76 subjects (average age 61.2 years; range 50–74 years) included 74 unifocal breast cancer patients and two cases of bilateral disease. All 78 of these pathologically proven non-palpable lobular cancers were enrolled and reviewed in this study. Anomalies were found in the right breast for 36/78 (46%) cases and in the left breast for 42/78 (54%) cases. The size of these lesions ranged 4–50 mm with a mean of 17 mm. The mammographic findings were distributed as follows: 50/78 (64%) opacity; 5/78 (6%) opacity plus calcifications, 21/78 (27%) architectural distortions, and 2/78 (3%) calcifications. DBT-VAB determined the biopsy route in 25/78 (32%) of these anomalies, while 53/78 (68%) lesions underwent ultrasound biopsy.

Breast parenchyma densities included fatty breasts (*n* = 57; 75%) and dense breasts (*n* = 19; 25%).

**Figure 1:**
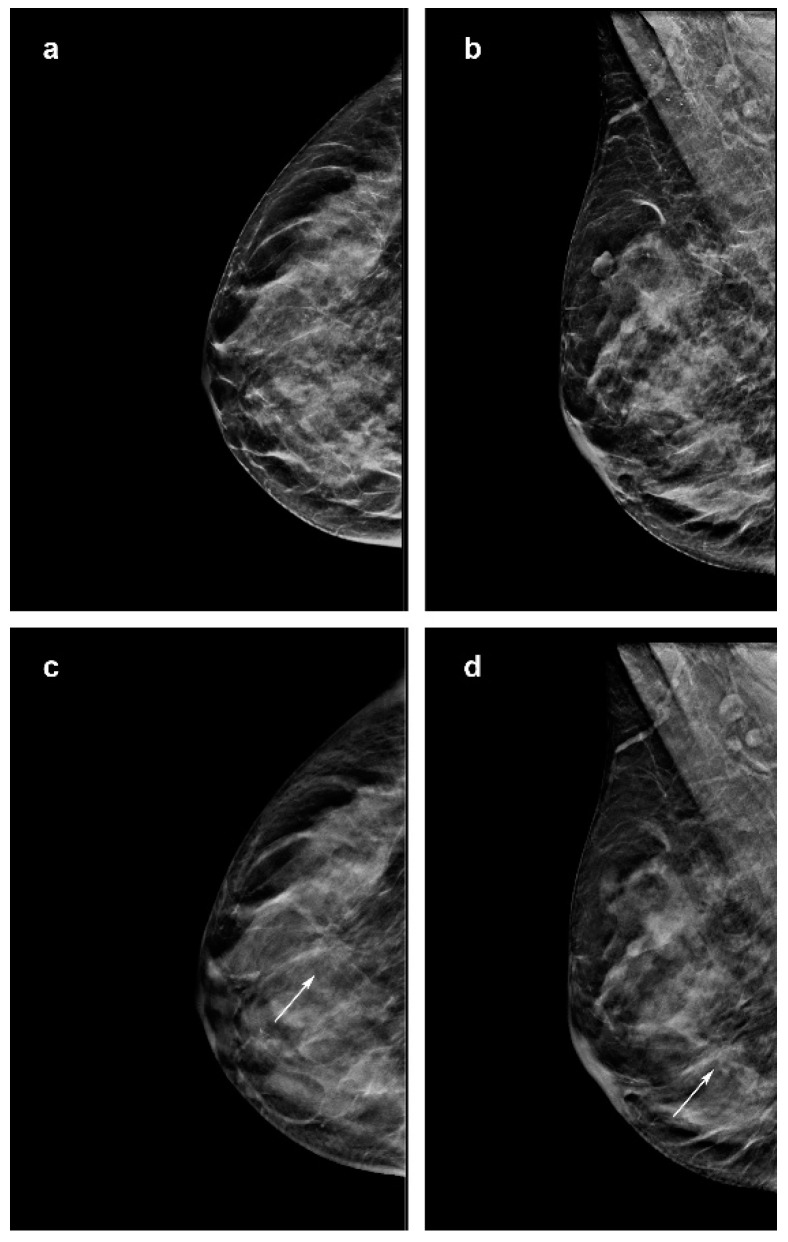
Images of a 56-year-woman with an invasive lobular carcinoma diagnosed at screening digital breast tomosynt 20 mm in the outer inferior quadrant (arrow) (**a–c**). (**d**) It is further less defined in the mediolateral oblique images of the DBT (arrow).

In our study, 50/78 (64%) cancers were detected on both synt2D and DBT by all the readers. While, 28/78 (26%) cancers were not recognized by at least one reader on synt2D, 4/78 (3.8%) cancers were missed by at least one reader on DBT. Opacity (34/50; 68%) and architectural distortions (10/50; 20%) represented the more evident mammographic signs. Opacity with calcifications was identified in 5/50 (10%) cases; while just one patient presented calcification. The subjective breast density ratings distributions of these anomalies were 38/50 (76%) and 12/50(24%) in fatty breasts and dense breasts, respectively. Moreover, 4/21 (19%) architectural distortions in dense breasts were only seen on DBT, by all the readers (Figure 1).

The lesion size distribution does not differ significantly from the normal distribution. The value of the Kolmogorov–Smirnov test statistic is 0.23761 (*p*-value 0.05653), with a mean of 21.77 and standard deviation of 5.1, respectively.

**Figure 2:**
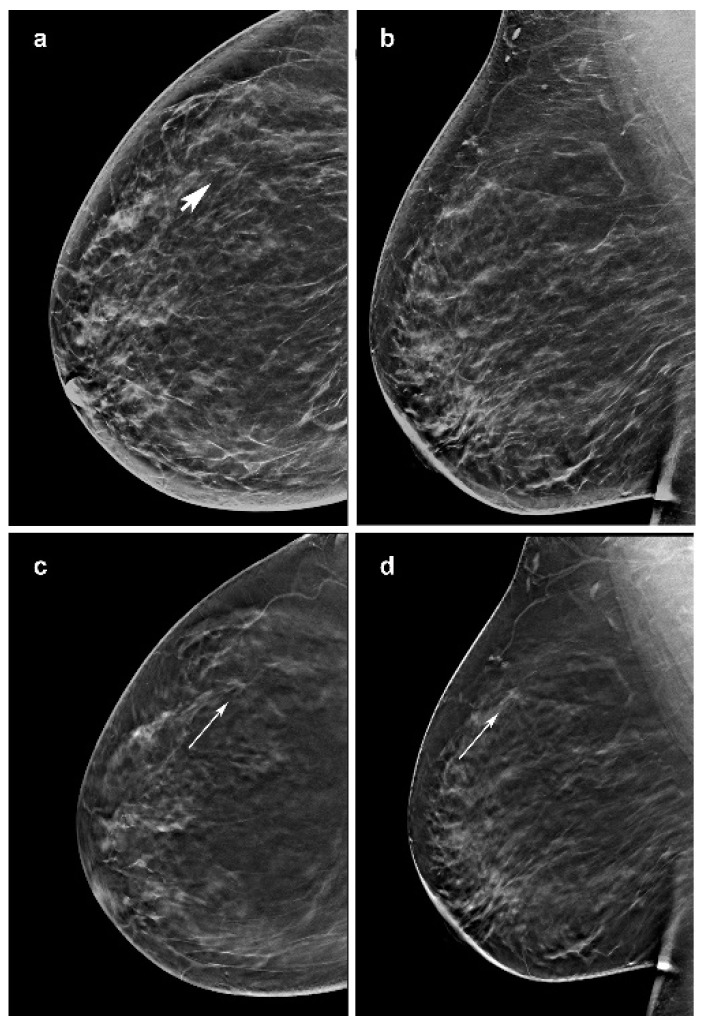
Invasive lobular carcinoma presenting as a mass in a 57-year-woman, diagnosed at screening. (**c**,**d**) Craniocaudal and mediolateral oblique images of the right breast from slices of the DBT portion of the screening study demonstrate a poorly defined 7-mm mass in the outer superior quadrant (arrow). (**a**) It is less defined in the craniocaudal synthesized mammography (arrow head) and not visible in the mediolateral oblique synthetized mammography oblique image (**b**).

All but one of these lesions were grade II and the remaining tumors presented grade I. One architectural distortion of 12 mm in a dense breast was just recognized in DBT by three readers and three architectural distortions in fatty breast were seen only in DBT by all readers but one. In total, 2/50 (4%) opacities in fatty breast were only seen on DBT by all the readers, with a size respectively of 6 and 7 mm: both of grade I at histo-pathological analysis. Two opacities were not seen in synt2D by all the readers but one (respectively 10 mm in dense breast and 25 mm in fatty breast), one opacity of 6 mm in a fatty breast was not recognized in synt2D by three readers and five opacities were not recognized in synt2D by two readers. All these eight lesions were seen by all the readers on DBT (Figure 2). 

All five cases of opacity with calcifications were identified and the calcification lesions were seen by all the readers on both synt2D and DBT.

**Figure 3:**
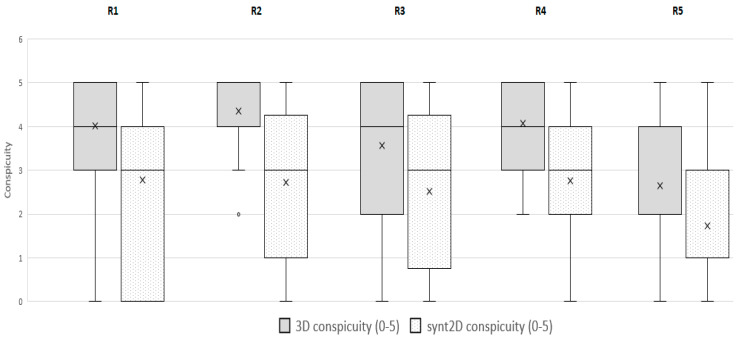
Reader-specific conspicuity. Bar graphs show reader-specific conspicuity evaluation stratified by imaging technique: digital breast tomosynthesis (DBT) or synthesized 2D mammograms (synt2D).

**Figure 4:**
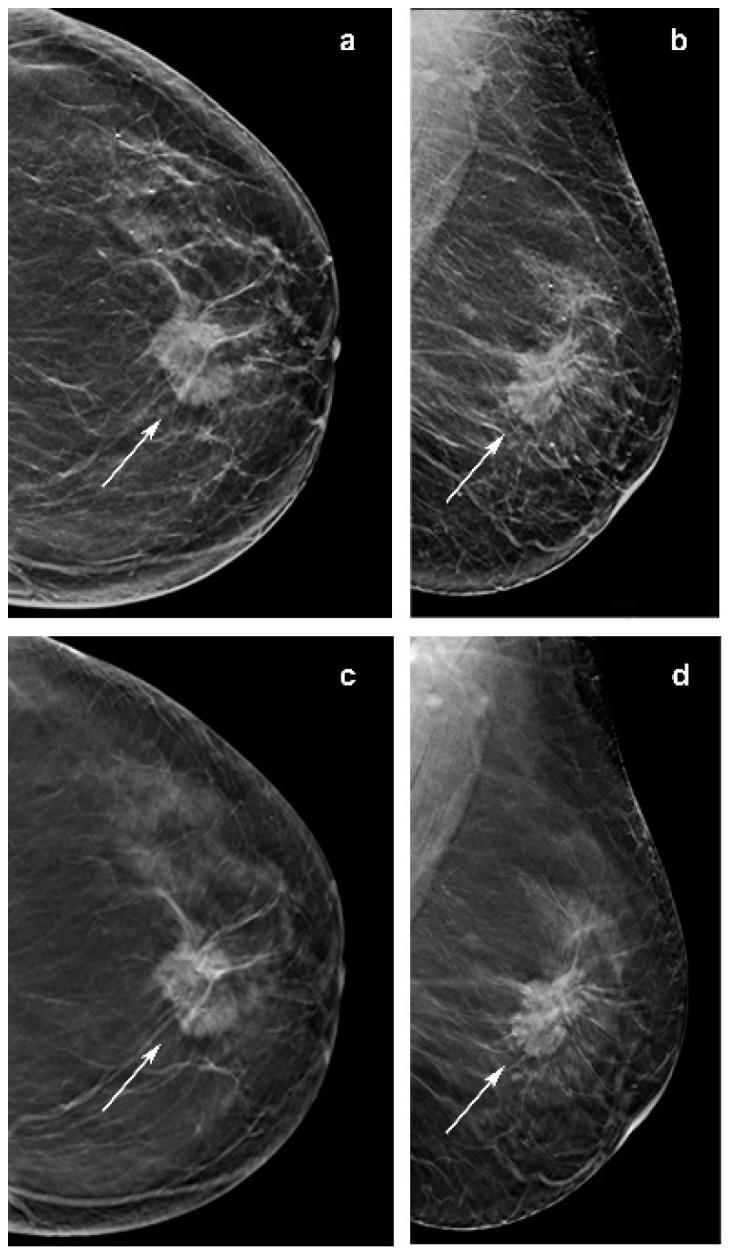
Images of a 65-year-woman with a 38 mm, spiculated and palpable mass (arrow in **a**–**d**) in the upper central quadrant of the left breast detected at screening with digital breast tomosynthesis (DBT) plus synthetic mammography (synt2D). Mass was invasive lobular carcinoma. (**c**) Image from single-slice DBT in craniocaudal view. (**d**) Single-slice DBT image in mediolateral oblique view. (**a**) Image from synt2D in craniocaudal view. (**b**) Image in mediolateral oblique synt2D view. The mass is easily identifiable in synt2D and DBT, but DBT (**c**,**d**) increases the contrast, emphasizing the presence of spicules, and resulting in a better estimate of size.

However, considering each single reader, 21/78 (27%) tumors were detected with only DBT for readers 1; 14/78 (18%) for reader 2; 19/78 (24%) for reader 3; 13/78 (17%) for reader 4 and 14/78 (18%) for reader 5. For each reader in comparison with synt2D, DBT significantly increased the conspicuity of ILC (*p* < 0.0001; results are shown in Figure 3) (Figure 4). 

**Figure 5:**
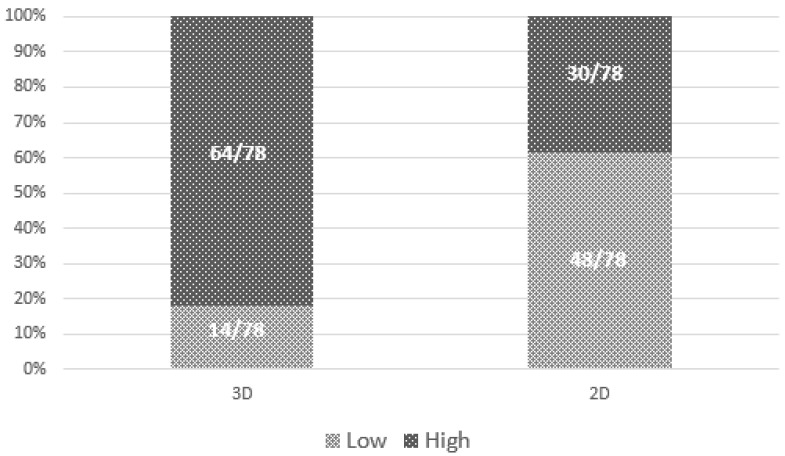
Distribution of high versus low conspicuity. Bar graphs demonstrate the conspicuity distribution of lesions (low conspicuity or high conspicuity), comparing digital breast tomosynthesis (DBT) or synthesized 2D mammograms (synt2D).

Figure 5 shows the raw proportions of high versus low conspicuity by modality, confirming that cancers were more likely to have high conspicuity at DBT than at synt2D.

At definitive histology, the tumor size of the lobular carcinoma was pT1 in 57/76 cases (75%), pT2 in 18/76 cases (23.68%), and pT3 in 1/76 cases (1.32%). One patient had lymph node positive disease.

## 4. Discussion

In recent years there has been consistent evidence that synthesized mammography (synt2D) in place of conventional mammography in screening and symptomatic settings is at least the equal of standard 2D digital mammography in the detection of malignancy [6,7,8,9,10].

However, the literature confirmed that the use of digital breast tomosynthesis (DBT) in combination with synthesized mammography (synt2D) still increases the accuracy of the detection of malignancy across age-groups and breast density [11].

Previous studies reported the benefit of DBT compared with DM for breast cancer screening [3]; however, relatively little is known about differences in cancer conspicuity between the two modalities. 

In studies of radiographic breast lesions, conspicuity is defined as the lesion contrast divided by the surrounding complexity. Therefore, we decided to investigate specific differences in breast cancer conspicuity by mammographic modality (DBT versus synt2D) in invasive lobular carcinoma (ILC), because this tumor is difficult to detect by mammogram due to its peculiar growth pattern, unique to the lesion [12].

As shown by the literature, our study confirmed that DBT could increase the conspicuity of ILC (Figure 3). In fact, 4/78 lesions were not recognized using only synt2D and were recovered by all breast dedicated readers only in DBT. Moreover, 18 to 27% of cases were recovered with DBT by at least one reader. Our data indicate that DBT increases detection of lobular breast cancer compared with synt2D: ILCs were more likely to have high conspicuity at DBT than at synt2D for each reader (Figure 5). 

The multi-reader study conducted by Mariscotti et al. [5] reported that DBT, as an adjunct to synt2D, significantly improved ILC detection. 

In our study, opacities and architectural distortions were the more evident mammographic signs (respectively 58% and 20%), in accordance with the literature [12,13].

Focusing on its histopathological architecture, ILCs usually extend in a single-file growth pattern, with little desmoplastic reaction and no hemorrhage or necrosis, that appear with specific features on imaging. Previous results supported that the use of DBT highlights architectural distortions defining lesion margins or spiculation [12]. When these contrast differences are small, mostly in dense breasts, our data confirm that the detection of ILC on mammography becomes increasingly difficult. In our series, 38% (8/21) of architectural distortions were difficult cases on imaging. All readers did not visualize four architectural distortions in dense breast on synt2D, although these lesions were visible on DBT. These architectural distortions are challenging for the breast radiologist, even regarding its diameter size, ranging between 15 to 30 mm, with a mean of 21 mm (Figure 1). While one architectural distortion of 12 mm in a dense breast was recognized in 3D DBT by only three readers, three architectural distortions in fatty breast were seen in DBT by all the readers but one. These data are in line with several studies [12] showing that DBT has better capabilities than synt2D in lesion detection and characterization; removing the tissue above and below the plane of the lesion means the size and margins can be more readily assessed. 

Other investigations reported that ILC is commonly presented as a mass on mammography. Of note, in our series 10/50 opacities were not recognized by at least one reader at synt2D but were visualized by all the readers at DBT. Two out of ten tumors with small diameter were identified in fatty breast only at DBT by all the readers (Figure 2). This could be explained by the better capabilities of DBT than synt2D in the evaluation of spiculated or ill-defined masses (Figure 3). Our results confirmed that calcifications with or without opacity due to their high density are easy to detect on both synt2D and DBT.

Previous studies about ILC size at diagnosis reported that about 40% [14] of ILCs were pT2+, while in our study the percentage of pT2+ was only 25%. Therefore, DBT can improve early diagnosis of ILC. 

Our study was limited by its retrospective design, and all readers were aware that all mammograms had an anomaly corresponding to ILC. Furthermore, all patients were derived from our practice, and readers were from our institution.

In conclusion, DBT increases ILC conspicuity, improving early diagnosis and detection of this type of cancer. Further studies are necessary with higher numbers, a multi-center approach, and with an implemented dataset to confirm our results.

## Data Availability

The data presented in this study are available on request from the corresponding author. The data are not publicly available due to privacy.
